# Protective Effects of Apamin on Acetaminophen-Induced Hepatotoxicity in Mice

**DOI:** 10.3390/cimb45050279

**Published:** 2023-05-17

**Authors:** Hyo-Jeong Jang, Jaechan Leem, Gyun Moo Kim

**Affiliations:** 1 Department of Pediatrics, School of Medicine, Keimyung University, Daegu 42601, Republic of Korea; polarisjay@dsmc.or.kr; 2 Department of Immunology, School of Medicine, Daegu Catholic University, Daegu 42472, Republic of Korea; 3 Department of Emergency Medicine, School of Medicine, Daegu Catholic University, Daegu 42472, Republic of Korea

**Keywords:** acetaminophen, apamin, hepatotoxicity

## Abstract

Acetaminophen (APAP) overdose can cause severe liver damage, but therapeutic options are limited. Apamin is a natural peptide present in bee venom and has antioxidant and anti-inflammatory properties. Accumulating evidence suggests that apamin has favorable actions in rodent models of inflammatory disorders. Here, we examined the effect of apamin on APAP-evoked hepatotoxicity. Intraperitoneal administration of apamin (0.1 mg/kg) alleviated histological abnormalities and reduced serum levels of liver enzymes in mice injected with APAP. Apamin inhibited oxidative stress through an increase in the amount of glutathione and activation of the antioxidant system. Apamin also attenuated apoptosis with inhibition of caspase-3 activation. Moreover, apamin reduced serum and hepatic levels of cytokines in APAP-injected mice. These effects were accompanied by suppression of NF-κB activation. Furthermore, apamin inhibited chemokine expression and inflammatory cell infiltration. Our results suggest that apamin dampens APAP-evoked hepatotoxicity through inhibiting oxidative stress, apoptosis, and inflammation.

## 1. Introduction

Drug-induced liver injury (DILI) can result from taking various drugs, such as chemical agents, biologics, herbs, or other xenobiotics [1,2]. Accumulating evidence suggests that DILI is closely associated with substantial mortality [1]. Acetaminophen (APAP) is a medication commonly used to reduce fever and pain. APAP overdose is a frequent cause of DILI and has become a global health problem [3]. After taking APAP, about 90% is metabolized to non-toxic metabolites, but the remainder is metabolized to N-acetyl-p-benzoquinone imine (NAPQI) [4]. This metabolite is detoxified by conjugating with glutathione (GSH). APAP overdose leads to GSH depletion and the accumulated NAPQI causes oxidative stress [4]. Oxidative stress induced by NAPQI can cause hepatocyte death and inflammation, leading to liver damage [5]. N-acetylcysteine is the antidote for APAP poisoning and supplements GSH to detoxify NAPQI [6]. However, its use is limited owing to the low efficacy of its delayed use and the narrow therapeutic window [5]. Thus, it is necessary to develop new drugs for APAP-induced hepatotoxicity.

Bee venom has been used to treat numerous medical conditions, including arthritis, musculoskeletal pain, and multiple sclerosis, in traditional medicine [7]. This natural product is composed of various bioactive compounds, including proteins, peptides, and other low-molecular components [7]. Among them, apamin (APM) is one of the main bioactive components of bee venom [8]. This peptide is composed of 18 amino acids and is known to inhibit small-conductance calcium-activated potassium (SK) channels [9]. These channels play a critical role in learning and memory by modulating synaptic plasticity [10]. APM has been shown to regulate learning and memory functions by blocking SK channels [11,12]. Over the years, many studies have focused on the action of APM as a SK channel blocker in learning and memory deficits [9]. However, emerging evidence suggests that APM also exerts other biological actions, such as antioxidant and anti-inflammatory activities [13]. Previous studies have reported that APM ameliorates various inflammatory diseases, including acute kidney injury [14], gouty arthritis [15], acute pancreatitis [16], and atherosclerosis [17] in rodents. In addition, APM alleviated inflammatory and fibrotic processes in mouse models of chronic liver disease [18,19]. However, whether APM ameliorates APAP-evoked hepatotoxicity remains unclear. This study aimed to investigate the effect and mechanism of action of APM on APAP-evoked hepatotoxicity.

## 2. Materials and Methods

### 2.1. Animal Study

Male C57BL/6N mice (8 weeks old; HyoSung Science, Daegu, Republic of Korea) were arbitrarily divided into 3 groups (n = 8/group): the Control group, the APAP group, and the APAP+APM group. After fasting for 12 h, the APAP and the APAP+APM groups received an intraperitoneal injection of APAP (400 mg/kg; Sigma-Aldrich, St. Louis, MO, USA). In the APAP+APM group, APM (0.1 mg/kg; dissolved in sterile saline; Sigma-Aldrich, St. Louis, MO, USA) was intraperitoneally administered 1 h after APAP treatment. The APAP group was given an equal volume of saline. Mice were sacrificed 24 h after APAP treatment. The doses of APM and APAP were chosen based on the published literature [14,20]. The Institutional Animal Care and Use Committee of the Daegu Catholic University Medical Center approved the research protocol (DCIAFCR-201106-12-Y).

### 2.2. Biochemical Analysis

An automatic analyzer (Hitachi, Osaka, Japan) was used for analyzing serum aspartate aminotransferase (AST) and alanine aminotransferase (ALT) levels. Hepatic malondialdehyde (MDA) concentrations were assessed using the MDA assay kit (Sigma-Aldrich, St. Louis, MO, USA). Serum 8-hydroxy-2′-deoxyguanosine (8-OHdG) levels were analyzed using the 8-OHdG assay kit (Abcam, Cambridge, MA, USA). Hepatic levels of GSH and oxidized GSH (GSSG) were assessed using the GSH detection kit (Enzo Life Sciences, Farmingdale, NY, USA). Serum levels of tumor necrosis factor-α (TNF-α), interleukin-6 (IL-6), and IL-1β were determined using ELISA kits (R&D Systems, Minneapolis, MN, USA). Hepatic myeloperoxidase (MPO) activities were measured using the MPO activity assay kit (Abcam, Cambridge, MA, USA).

### 2.3. Histological Analysis, Immunochemistry (IHC), and Immunofluorescence (IF) Staining

Liver tissues were fixed, paraffin-embedded, and sectioned. The slides were stained with hematoxylin and eosin (H&E) stain. The percentage of necrotic area was determined in 10 randomly selected fields (200×) per sample.

The sections were deparaffinized, rehydrated, and stained with antibodies against 4-hydroxynonenal (4-HNE; Abcam, Cambridge, MA, USA) or F4/80 (Santa Cruz Biotechnology, Dallas, TX, USA) for IHC. After being washed, the sections were incubated with a secondary antibody. The percentage of 4-HNE-stained area and the number of F4/80-positive cells were determined in 10 randomly chosen fields (200× for 4-HNE, 400× for F4/80) per sample. Primary antibodies against 8-OHdG (Santa Cruz Biotechnology, Dallas, TX, USA) or Ly6B.2 (Abcam, Cambridge, MA, USA) were used for IF staining. Positive cells were counted in 10 randomly chosen fields (600×) per sample.

### 2.4. Terminal Deoxynucleotidyl Transferase-Mediated dUTP Nick-End Labeling (TUNEL) Staining

Apoptosis was analyzed using a TUNEL assay kit (Roche Diagnostics, Indianapolis, IN, USA). Positive cells were counted in 10 arbitrarily chosen fields (600×) per sample.

### 2.5. Western Blot Analysis

Total proteins were extracted using a RIPA lysis buffer. The samples were loaded onto polyacrylamide gels and transferred to nitrocellulose membranes. The membranes were incubated with the following primary antibodies: cleaved caspase-3, cleaved poly(ADP-ribose) polymerase-1 (PARP-1), nuclear factor-κB (NF-κB) p65, p-NF-κB p65, and glyceraldehyde-3-phosphate dehydrogenase (GAPDH). All primary antibodies were purchased from Cell Signaling (Danvers, MA, USA). After being washed, the membranes were incubated with secondary antibodies. Protein bands were detected using enhanced chemiluminescence reagents (Thermo Fisher Scientific, Waltham, MA, USA).

### 2.6. Quantitative Reverse Transcription–Polymerase Chain Reaction (qRT-PCR)

Total RNA was extracted using TRIzol reagent. The PrimeScript RT Reagent Kit (TaKaRa, Tokyo, Japan) was used to reverse-transcribe the RNA into cDNA. Then, qRT-PCR was conducted using the Power SYBR Green PCR Master Mix (Thermo Fisher Scientific, Waltham, MA, USA) and primers (Table 1). Relative mRNA levels were normalized to GAPDH mRNA levels.

### 2.7. Statistical Analysis

Data are shown as mean ± SEM. Comparisons between groups were performed using one-way ANOVA analysis with Bonferroni’s test. A *p* value of <0.05 was considered statistically significant.

## 3. Results

### 3.1. APM Dampened APAP-Evoked Hepatotoxicity

**Figure 1:**
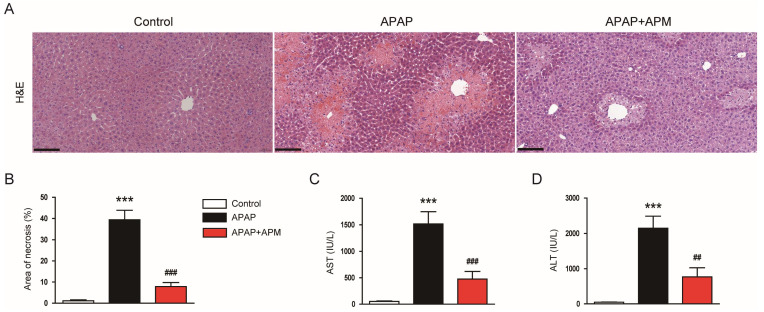
Effects of APM on histological abnormalities and liver enzymes in APAP-injected mice. (**A**) H&E staining. Scale bar: 100 μm. (**B**) Percentage of the necrotic area. (**C**) Serum AST levels. (**D**) Serum ALT levels. Data are shown as mean ± SEM. n = 8/group. *** *p* < 0.001 vs. Control. ^##^ *p* < 0.01 and ^###^ *p* < 0.001 vs. APAP.

To determine the effect of APM on APAP-evoked hepatotoxicity, we first performed H&E staining. As expected, APAP-injected mice exhibited increased necrotic area compared to control mice (Figure 1A,B). However, administration of APM reduced the necrotic area in APAP-injected mice (Figure 1A,B). Moreover, serum AST and ALT levels, liver injury indicators [27], were increased after APAP treatment (Figure 1C,D). APM reduced the levels of these indicators in APAP-injected mice (Figure 1C,D). These results show that APM has an ameliorative effect on APAP-induced hepatotoxicity.

### 3.2. APM Inhibited Oxidative Stress in APAP-Injected Mice

**Figure 2:**
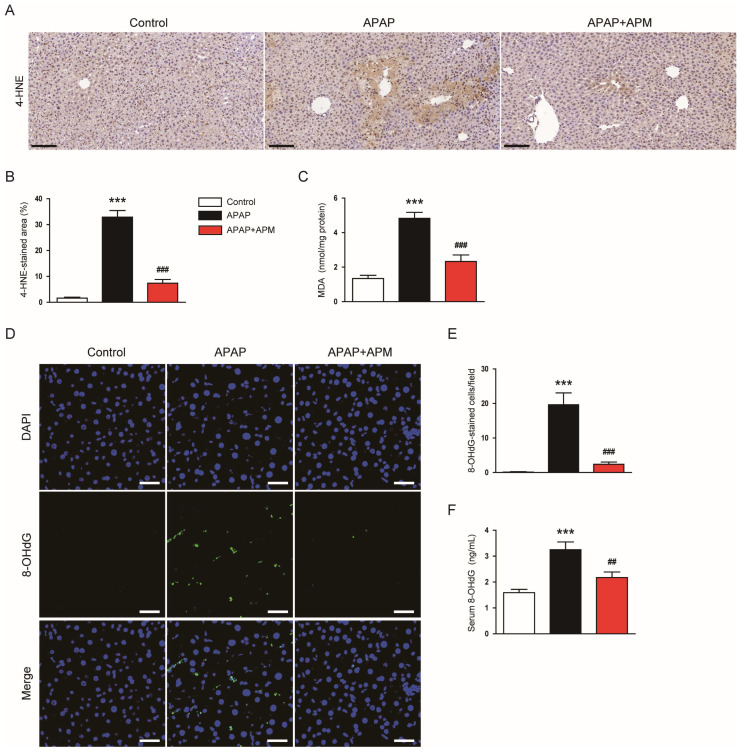
Effects of APM on oxidative stress. (**A**) IHC for 4-HNE. Scale bar: 100 μm. (**B**) Percentage of the 4-HNE-stained area. (**C**) MDA levels. (**D**) IF staining for 8-OHdG (green). Scale bar: 40 μm. (**E**) Number of 8-OHdG-stained cells per field. (**F**) Serum 8-OHdG levels. Data are shown as mean ± SEM. n = 8/group. *** *p* < 0.001 vs. Control. ^##^ *p* < 0.01 and ^###^ *p* < 0.001 vs. APAP.

To assess oxidative stress, we measured 4-HNE and MDA levels [28,29]. The area of 4-HNE staining increased after APAP injection (Figure 2A,B). However, APM remarkably suppressed 4-HNE expression (Figure 2A,B). Hepatic MDA concentrations were also decreased by APM (Figure 2C). Moreover, APM reduced the number of cells stained with 8-OHdG, a DNA oxidation marker [30], in APAP-injected mice (Figure 2D,E). APM also decreased serum 8-OHdG levels (Figure 2F).

**Figure 3:**
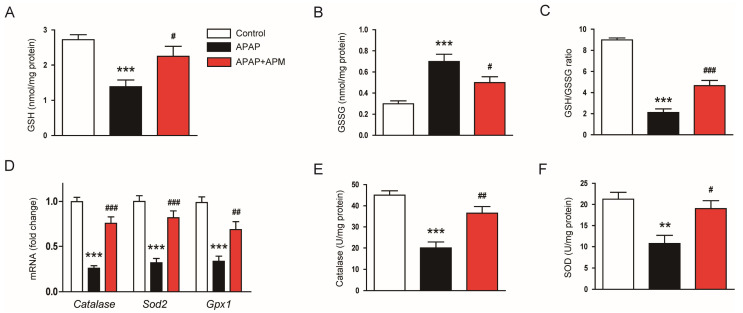
Effects of APM on redox status and antioxidant enzymes. (**A**) GSH levels. (**B**) GSSG levels. (**C**) GSH/GSSG ratios. (**D**) mRNA levels of *Catalase*, *Sod2*, and *Gpx1*. (**E**) Catalase activities. (**F**) SOD activities. Data are shown as mean ± SEM. n = 8/group. ** *p* < 0.01 and *** *p* < 0.001 vs. Control. ^#^ *p* < 0.05, ^##^ *p* < 0.01, and ^###^ *p* < 0.001 vs. APAP.

APAP injection decreased hepatic GSH levels (Figure 3A) and GSH/GSH ratios (Figure 3C) while increasing GSSG levels (Figure 3B). However, these changes were remarkably reversed by APM (Figure 3A–C). APM increased mRNA expression of *Catalase* (Figure 3D), *Sod2* (superoxide dismutase 2; Figure 3D), and *Gpx1* (glutathione peroxidase 1; Figure 3D) in APAP-injected mice. Reduced activities of catalase and SOD after APAP injection were also significantly recovered by APM (Figure 3E,F).

### 3.3. APM Suppressed Apoptotic Cell Death

**Figure 4:**
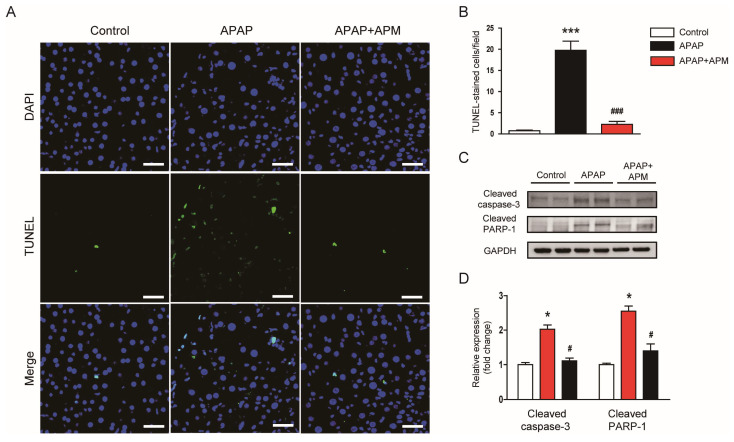
Effects of APM on apoptosis. (**A**) TUNEL staining. Scale bar: 40 μm. (**B**) Number of TUNEL-stained cells per field. (**C**) Western blotting of cleaved caspase-3 and cleaved PARP-1. (**D**) Quantification of protein bands in (**C**). Data are shown as mean ± SEM. n = 8/group. * *p* < 0.05 and *** *p* < 0.001 vs. Control. ^#^ *p* < 0.05 and ^###^ *p* < 0.001 vs. APAP.

Hepatocyte apoptosis plays a role in APAP-evoked hepatotoxicity [31,32]. The number of cells stained with TUNEL increased after APAP treatment (Figure 4A,B). However, APM remarkably suppressed the APAP-induced apoptosis (Figure 4A,B). Cleaved forms of caspase-3 (Figure 4C,D) and PARP-1 (Figure 4C,D) were also reduced by APM, indicating that the peptide inhibited caspase-3 pathway.

### 3.4. APM Suppressed Inflammatory Responses

**Figure 5:**
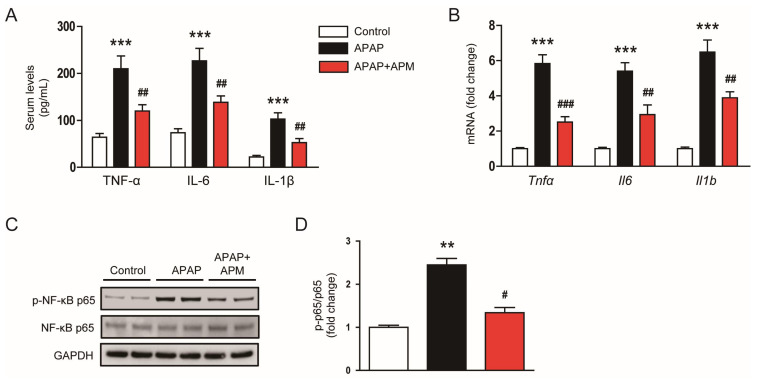
Effects of APM on cytokine generation and NF-κB activation. (**A**) Serum and (**B**) hepatic mRNA levels of *Tnfα*, *Il6*, and *Il1b*. (**C**) Western blotting of p-NF-κB p65. (**D**) Quantification of protein bands in (**C**). Data are shown as mean ± SEM. n = 8/group. ** *p* < 0.01 and *** *p* < 0.001 vs. Control. ^#^ *p* < 0.05, ^##^ *p* < 0.01, and ^###^ *p* < 0.001 vs. APAP.

Excessive inflammatory responses also serve a significant role in APAP-induced hepatotoxicity [5]. Elevated serum levels of TNF-α (Figure 5A), IL-6 (Figure 5A), and IL-1β (Figure 5A) were decreased by APM. The peptide also decreased hepatic mRNA levels of these cytokines (Figure 5B). Moreover, APM inhibited NF-κB p65 phosphorylation (Figure 5C,D).

**Figure 6:**
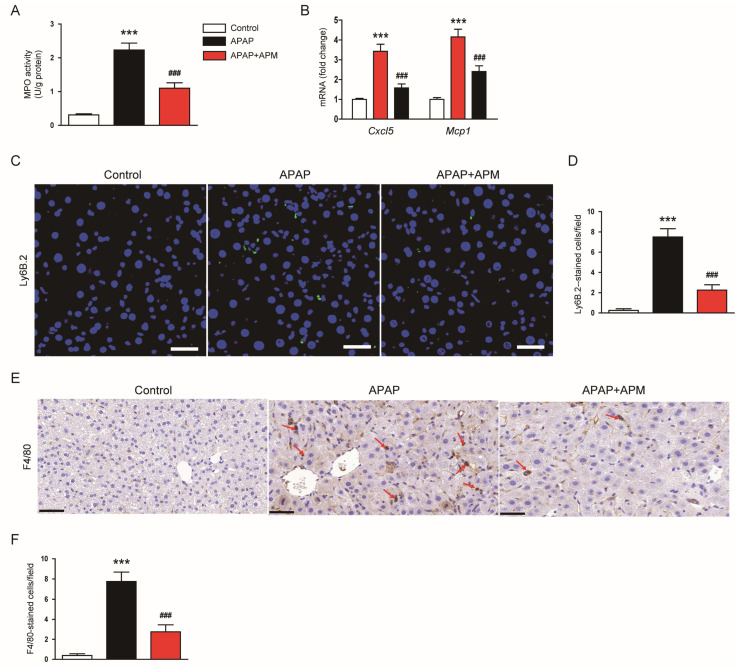
Effects of APM on inflammatory cell infiltration. (**A**) MPO activities. (**B**) mRNA levels of *Cxcl5* and *Mcp1*. (**C**) IF staining for Ly6B.2 (green). Scale bar: 40 μm. (**D**) Number of Ly6B.2-stained cells per field. (**E**) IHC for F4/80. Red arrows indicate positive cells. Scale bar: 50 μm. (**F**) Number of F4/80-stained cells. Data are shown as mean ± SEM. n = 8/group. *** *p* < 0.001 vs. Control. ^###^ *p* < 0.001 vs. APAP.

APAP treatment increases inflammatory cell infiltration, contributing to the aggravation of liver injury [33,34]. APAP-injected mice displayed increased activity of MPO, an abundant enzyme present in innate immune cells [35], in the kidney (Figure 6A). Hepatic MPO activity was remarkably inhibited by APM (Figure 6A). APM also reduced mRNA levels of *Cxcl5* (C-X-C motif chemokine ligand 5) and *Mcp1* (monocyte chemoattractant protein-1) in the liver (Figure 6B). The number of cells stained with Ly6B.2, a neutrophil marker [36], increased after APAP injection (Figure 6C,D). However, APM significantly inhibited neutrophil infiltration in APAP-injected mice (Figure 6C,D). IHC for F4/80 was also performed to identify macrophages [37]. APM remarkably reduced the number of F4/80-stained cells (Figure 6E,F).

## 4. Discussion

In this study, to assess the action of APM on APAP-evoked hepatotoxicity, we first performed histological analysis and measured serum levels of liver enzymes. When hepatocytes are injured, the plasma membrane becomes permeable, allowing the release of intracellular enzymes into the bloodstream. The two most commonly measured liver enzymes associated with clinical liver damage are AST and ALT. Therefore, measurement of serum levels of these enzymes is a good indicator of liver injury [38]. H&E staining showed that APAP-injected mice exhibited increased necrosis compared to control mice. Serum levels of the liver enzymes were increased after APAP injection. Importantly, administration of APM significantly reduced necrotic area and serum AST and ALT levels, suggesting that the peptide exerts a protective action on APAP-evoked hepatotoxicity.

APAP overdose causes GSH depletion and NAPQI accumulation. Excessive NAPQI induces oxidative stress, resulting in hepatocyte death and inflammation [5]. A previous study has demonstrated the production of NAPQI in the liver of C57BL/6N mice treated with APAP [39]. APM has been known to possess strong antioxidant property [13,14]. Therefore, we hypothesized that APM can ameliorate APAP-induced hepatotoxicity through inhibiting oxidative stress. In this study, APAP-injected mice exhibited GSH depletion and significant oxidative stress, as reflected by elevated amounts of lipid peroxidation and DNA oxidation products. However, administration of APM significantly restored GSH levels and attenuated oxidative damage in APAP-injected mice. APM significantly reversed the reduction in expression and activity of catalase and SOD2 in APAP-injected mice. These antioxidant enzymes protect the liver from oxidative damage in APAP-induced hepatotoxicity [40,41,42]. Previously, we showed that APM increased renal heme oxygenase-1 (HO-1) levels in mice injected with lipopolysaccharide (LPS) [14]. HO-1 is the enzyme responsible for breaking down heme into components with antioxidant properties [43]. Therefore, the HO-1 upregulation evoked by APM may be involved in its antioxidant and therapeutic effects on LPS-induced kidney injury. Altogether, our findings suggest that APM ameliorates oxidative stress via restoration of hepatic GSH depletion and upregulation of antioxidant enzymes in APAP-injected mice.

Necrosis is generally believed as the major mode of cell death in APAP-induced hepatotoxicity [4,5]. Although there are conflicting results [44], some studies suggest that apoptosis also plays a role in APAP-evoked hepatotoxicity [31,32]. Here, APAP-injected mice exhibited elevated numbers of TUNEL-positive apoptotic cells. However, APM remarkably inhibited hepatocyte apoptosis. Caspase-3 is an important effector caspase that cleaves various cytoplasmic and nuclear proteins, inducing apoptosis. This enzyme is activated by cleavage of the interdomain linker [45]. We found that APM reduced the cleavage of caspase-3 and its substrate PARP-1, indicating that the peptide inhibits caspase-3 activation. Consistent with our findings, APM protected macrophages from apoptosis evoked by oxidized low-density lipoprotein [46]. APM prevents loss of dopaminergic neurons through inhibiting the caspase-dependent mitochondrial apoptotic pathway [47]. Apoptosis can also be triggered by oxidative stress [5]. Therefore, inhibition of apoptosis by APM may be attributed, at least in part, to its antioxidant effect.

Inflammation also contributes to the development and progression of APAP-evoked hepatotoxicity [5]. Administration of APAP induced excessive cytokine generation and extensive inflammatory cell infiltration [33,34]. The pathogenic role of inflammatory responses was also supported by some human data [48,49,50]. In this study, APM decreased serum and hepatic concentrations of cytokines in APAP-injected mice, indicating that APM inhibits systemic and hepatic inflammation in APAP-induced hepatotoxicity. Moreover, APM inhibited NF-κB activation. This signaling cascade plays a pivotal role in APAP-evoked inflammatory responses [51,52]. Infiltration of neutrophils and macrophages was also decreased by APM. Consistently, APM reduced mRNA levels of chemokines (CXCL5 and MCP-1) in APAP-injected mice. Similar to our data, many researches have shown the anti-inflammatory effects of APM [13]. APM inhibited NF-κB cascade and cytokine secretion in LPS-treated microglial cells [53]. The peptide also reduced the generation of cytokines with the attenuation of NF-κB activation in inflamed human keratinocytes [54]. Lee et al. showed that APM treatment inhibited proinflammatory cytokine production and inflammasome activation in mice with gouty arthritis [15]. APM also reduced cytokine concentrations and MPO activity in a mouse model of acute pancreatitis [16]. In addition, APM inhibited cytokine production, NF-κB cascade, and inflammatory cell infiltration in endotoxemic mice [14].

In this study, we administered APM to mice 1 h after an APAP overdose. Therefore, this study has limitations in not reflecting a typical clinical situation in which several hours to several days are required for treatment after an APAP overdose. Similar to our study, however, in most previous animal studies, bioactive molecules were administered before or within 3 h of APAP administration [55,56,57]. Indeed, the injury process is known to progress much faster in mice than in humans after an APAP overdose [58]. Nonetheless, further experiments administering APM at longer time intervals after an APAP overdose will be needed to assess its applicability in the clinical setting.

In summary, we showed that APM has a therapeutic action against APAP-evoked hepatotoxicity in mice. APM attenuated oxidative stress via restoration of GSH depletion and upregulation of antioxidant enzymes. Hepatocyte apoptosis and caspase-3 activation was also alleviated by APM. Moreover, APM suppressed cytokine production, NF-κB activation, and inflammatory cell infiltration. We propose that APM might be a potential therapeutic option for APAP toxicity.

## Figures and Tables

**Table 1:** List of primers.

Gene	Primer Sequence (5′→3′)	Reference
*Catalase*	F: GCTGAGAAGCCTAAGAACGCA R: CCTTCGCAGCCATGTGAGA	[21]
*Sod2*	F: GCGGCCTACGTGAACAATCT R: CATCTCCCTTGGCCAGAGC	[21]
*Gpx1*	F: TTTCCAGGAAAATCCCCTCA R: TAGGTGGAAAGGCATCGGC	[21]
*Tnfα*	F: GTTCTGTCCCTTTCACTCACTG R: GGTAGAGAATGGATGAACAC	[22]
*Il6*	F: CCGGAGAGGAGACTTCACAG R: CAGAATTGCCATTGCACAAC	[23]
*Il1b*	F: TGGTGTGTGACGTTCCCATTA R: CCGACAGCACGAGGCTTTT	[21]
*Cxcl5*	F: TTGATCGCTAATTTGGAGGTG R: GCATTCCGCTTAGCTTTCTTT	[24]
*Mcp1*	F: ACTCACCTGCTGCTACTCATTCAC R: AACTACAGCTTCTTTGGGACACCT	[25]
*Gapdh*	F: CCCCAGCAAGGACACTGAGCAA R: GTGGGTGCAGCGAACTTTATTGATG	[26]

## Data Availability

Data are included in the article.
